# Dead regions in the cochlea at high frequencies: implications for the adaptation to hearing aids

**DOI:** 10.1016/S1808-8694(15)30072-0

**Published:** 2015-10-19

**Authors:** Angela Gordo, Maria Cecília Martinelli Iório

**Affiliations:** a Medical doctor, speech therapist, Doctor on The Science of Human Communication Disorders, trained at the Sao Paulo Federal University - Paulista School of Medicine; b Lecturer, Teacher in the discipline The Science of Human Communication Disorders at the Sao Paulo Federal University - Paulista School of Medicine. Head of the Discipline of Hearing Disorders at the Sao Paulo Federal University - Paulista School of Medicine

**Keywords:** speech perception, self-assessment, hearing aids, sensorineural hearing loss

## Abstract

In patients with moderate to severe high-frequency hearing loss, cochlear damage may include “dead regions” where there are no functional inner hair cells and/or associated neurons. **Aim:** This study examines speech recognition in sensorineural impaired hearing patients with and without cochlear dead regions at high frequencies. **Methods:** a clinical and experimental study was made of thirty patients with sensorineural hearing loss that were classified into two groups: group 1 - included 15 subjects with hearing loss and no dead regions; and group 2 - included 15 subjects with dead regions in the cochlea at high frequencies. Patients undertook word recognition score and speech reception threshold tests, with and without background noise. The speech tests were done with and without hearing aids in two situations: program 1 - broadband amplification (bandwidth 8000 Hz); and program 2 - amplification up to 2560 Hz, without high frequency gain. **Results:** For subjects with no dead regions in the cochlea (group 1) performance improved with program 1. For subjects with dead regions in the cochlea (group 2) performance improved with program 2. **Conclusions:** Subjects with no dead regions in the cochlea benefited from high-frequency information. Subjects with dead regions in the cochlea benefited from reduced gain at high frequencies.

## INTRODUCTION

In audiology, sloping auditory sensorineural deficiency is the most common type/configuration of hearing loss associated with difficulty in understanding speech in noisy environments. Although hearing aids may increase the available acoustic information, not always a satisfactory improvement in speech recognition is attained. Some patients enjoy little or not benefit from amplification, particularly in cases of sloping hearing loss where a severe grade occurs at high frequencies.

An old concept is the relation between absence of benefit from hearing aids and functional reduction and complete loss of inner hair cells and/or neurons in certain regions of the cochlea. No clinical test, however, was done to identify the dead zones in the cochlea.

In these regions information generated by vibration of the basilar membrane is not transmitted to the central nervous system. If sufficiently intense, however, a tone with a corresponding frequency to that of the dead zone may be detected through apical or basal transmission of the vibration pattern by other functional regions of the cochlea. The vibration amplitude of the basilar membrane at a distant site will be lower than the dead zone amplitude. Broad band noise may thus mask that tone much more effectively than expected, as noise needs only to eliminate the response coming from the remote site. If the threshold needed to detect a tone in the presence of broad band noise is higher than that of the normal threshold, this alteration may indicate lack of inner hair cells and/or adjacent neurons, with a typical frequency that corresponds to the tone frequency, in other words, a dead zone.^1^

A few studies have related the difficulty that hearing loss imposes on speech recognition to the need for hearing speech at high sound pressure levels; these levels, however, may reduce the analytical capability of the normal cochlea. As hearing loss increases, certain frequencies do not support or even reduce the available information at other frequencies. Less amplification should therefore be prescribed for frequencies at with auditory thresholds are increased.^2^, ^5^

Auditory resolution is the ability that inner ear structures and their associated neural systems have of generating patterns of neural activity that reflect spectral and time differences between sound information. The auditory nerve is organized, as is the basilar membrane: typically high frequency fibers originate from hair cells of the base of the cochlea, while low frequency fibers are at the apex of the cochlea. Fibers in the basal region respond synchronically to the presentation of a stimulus. Fibers in the apex are activated later (2 to 4ms later). Those that are activated simultaneously will contribute the most to the action potential of the whole nerve. Consequently this potential reflects mainly the response of high frequency fibers.^3^

Other authors4 have proposed a clinical test to identify dead zones of the cochlea; this test is the threshold equalizing noise (TEN), which compares auditory thresholds investigated with and without ipsilateral masking. The TEN noise level has a spectrum that was elaborated to obtain equally masked thresholds at all frequencies, (125 Hz to 15000 Hz), and is expressed as ERB (Equivalent Rectangular Bandwidth), which refers to the bandwidth of the auditory filter. Normal listeners have a small variation (2 dB to 3 dB) between masked thresholds and noise levels. In patients with sensorineural hearing loss, dead zones of the cochlea are found when masked thresholds are at least 10 dB over absolute thresholds and 10 dB over the noise level. The results are confirmed by the measurement of psychophysical tuning curves. When the TEN test is positive, the peak of the tuning curve is displaced relative to the signal frequency. The authors emphasize that if dead zones are present, it may be useful to amplify a frequency range slightly above the dead zone; the reason being that amplification should aim at where hair cells can make use of it.

The effectiveness of hearing for intelligibility is affected by the sound pressure level of the signal (distortion level), the degree of hearing loss, the frequencies at which it takes place, and the sound information processing capability.^5^ As thresholds increase, the auditory efficiency decreases; this effect is amplified in high frequency hearing loss. The practical implication of this concept for hearing aid adaptation is that increased amplification should be applied where thresholds are less affected.

Based on these thoughts, the aim of this study was to verify the benefit of high frequency amplification for speech recognition in patients with sloping sensorineural hearing loss with or with no dead zones in the cochlea.

## METHODS

The procedures used in this study were described to the Research Ethics Committee and approved under code number 0235/04. Participants signed a free informed consent form containing the necessary information before undergoing the tests. We assessed 30 subjects (14 women and 16 men) with sloping, bilateral, and symmetrical sensorineural hearing loss. Identification of dead zones of the cochlea was done using the TEN(NA) test,6 2004 version. Two groups were defined based on these results: group 1 - 15 subjects with no dead zones of the cochlea, and group 2 - 15 subjects with dead zones of the cochlea at high frequencies.

Tables 1 and 2 show the distribution of the sample population including gender, age, complaint, duration of the complaint, and use of hearing aids (if a user for how long or if in the selection/adaptation process) for group 1 and 2.

**Table 1:** Distribution of the sample population as to gender, age, complaint, duration of complaint, and use of hearing aids in group 1.

Sex	Age	Complaint	Duration	Hearing aids
M	45	Sudden hearing loss	40 years	8 months
F	49	Listens but does not understand speech	3 years	Adapting
M	49	Progressive hearing loss / tinnitus	6 years	1 year
M	58	Progressive hearing loss / tinnitus	3 years	Adapting
M	66	Progressive hearing loss / tinnitus	10 years	1 year
M	68	Progressive hearing loss	6 years	6 years
M	69	Progressive hearing loss	2 years	1 month
M	71	Progressive hearing loss / tinnitus	6 years	Adapting
F	72	Progressive hearing loss	5 years	Adapting
M	73	Progressive hearing loss	3 years	Adapting
M	74	Does not understand speech	20 years	3 months
F	75	Progressive hearing loss	10 years	3 months
M	75	Progressive hearing loss	22 years	4 months
F	76	Progressive hearing loss	2 years	1 month
M	83	Does not understand speech / tinnitus	20 years	1 year

**Table 2:** Distribution of the sample population as to gender, age, complaint, duration of complaint, and use of hearing aids in group 2.

Sex	Age	Complaint	Duration	Hearing aids
F	19	Would like to hear better	16 years	1 year
F	24	Does not understand speech / tinnitus	12 years	Adapting
F	26	Does not understand speech	6 years	Adapting
F	34	Does not understand speech	10 years	Adapting
M	35	Does not hear/discomfort for loud sounds	10years	Adapting
F	36	Does not understand speech	12 years	9 months
F	43	Progressive hearing loss / tinnitus	4 years	1 mês
M	50	Constant tinnitus	3 years	4 months
M	54	Does not hear well / tinnitus	5 years	Adapting
M	57	Does not hear well / tinnitus	10 years	Adapting
M	64	Progressive hearing loss / tinnitus	20 years	Adapting
F	69	Progressive hearing loss / tinnitus	8 years	2 months
F	73	Does not understand speech / tinnitus	15 years	Adapting
F	75	Does not understand speech / tinnitus	30 years	1 month
F	75	Does not hear well / does not understand speech	20 years	2 years

The TEN test was applied using a two channel audiometer to control separately the stimulus (pure tone) and noise. The audiometer was coupled to a CD player. Auditory thresholds were measured in 2 dB intervals at 500, 750, 1000, 1500, 2000, 3000, and 4000 Hz in each ear separately, using TDH49 earphones, initially without masking, followed by an ipsilateral TEN noise at 70 dB NA/ERB. If this level was not sufficient to mask the absolute threshold, we would gradually increase the intensity up to 85 dB NA/ERB (maximum noise level tolerated by our patients). When the masked threshold was 10 dB or more over the absolute threshold and the noise level, we would give the result as suggesting dead zones for the tested frequency.

We then proceeded with speech recognition tests in quite and with background noise, presenting the stimuli in an acoustic room, always using the same loudspeaker that the patient sat facing at a distance of 1 meter and azimuth 0º.

We used a digital Siemens Signia HdO+ behind the-ear hearing aid with eight independently programmable channels for different frequency ranges to observe performance according to the amplified signal. Adaptation of hearing aids was binaural in all subjects. There were two programs: program 1 was amplification at a wide range of low frequencies (between 100 Hz and 8000 Hz), and program 2 had no gain at high frequencies (over 2560 Hz). The cut-off frequency for program 2 was approximately 2000 Hz, which was selected based on research that reports benefits from amplification of 1.7 times the dead zone limit frequency (roughly one octave higher).^7^ Although this measurement was not done precisely in our study, results were positive for dead zones starting between 1000 and 1500 Hz in most cases.

The speech material used for the Percentage Index of Speech Recognition (PISR) survey was a list of 25 phonetically balanced monosyllables^8^ recorded in four difference sequences. The PISR was assessed in quite and with background speech noise. With no hearing aids, speech intensity was set as the most comfortable level reported by patients. With hearing aids, speech and noise were set at 65 dB A. We then investigated the Sentence Recognition Threshold in quiet (SRTQ) and in the presence of background noise (SRTN) using five lists containing 10 phonetically balanced sentences.9 For the SRTQ test we presented the first sentence using the best ear speech reception threshold (obtained by earphones). Noise was set at 65 dB A for the SRTN test, and the first sentence was presented always in a zero signal-to-noise ratio. Both tests (PISR and SRTQ/N) were investigated under three different conditions: unaided, aided using program 1, and aided using program 2; the sequence of procedures and the selected lists were alternated at each presentation.

We then applied the Abbreviated Profile of Hearing Aid Benefit (APHAB)^10^ questionnaire to assess communication difficulties in daily situations. This questionnaire includes 24 items on three communication subscales related to the acoustic environment and one subscale on discomfort for intense sounds, namely: ease of communication, reverberation, background noise and aversiveness of sounds. As not all subjects used hearing aids, we used only the responses for unaided performance. Participants chose the option for each item that came closest to their everyday experience: A-always (99%); B-almost always (87%); C-generally (75%); D-half-the-time (50%); E-occasionally (25%); F-seldom (12%); G-never (1%). Results were quantified to reach a score for each subscale.

We used non-parametric comparison tests to analyze our results statistically. Since the sample was relatively small, our significance level was set at 0.07 (7%).

Masked thresholds were never over 10 dB above the noise level (from 70 to 85 dB NA/ERB) in all patients; the maximum difference between them was 6 dB NA. Generally only one noise level (70 dB NA/ERB) was enough for the TEN(NA) test. When the audiometric threshold was above 60 dB at a specific frequency, we used 10 dB over this threshold to define the minimum masking level.

When masked auditory thresholds obtained by TEN(NA) testing of group 2 exceeded one or more of the absolute thresholds and the noise level by 10 dB, we considered the result as positive for dead zones of the cochlea at high frequencies. We used two or three noise levels due to the degree of hearing loss at these frequencies. Various patients had a 10 dB or more change compared to the absolute threshold when the noise level was below this threshold, where masking would theoretically be insufficient to change the threshold.

In subjects with no dead zones of the cochlea we found that the PISR in quiet and in the presence of background noise improved significantly when using program 1 compared to the unaided and aided conditions of program 2.

When dead zones of the cochlea were present we found that the PISR in quiet and in the presence of background noise improved significantly when using programs 1 and 2 in unaided conditions, and that program 2 showed significantly improved results compared to program 1.

SRTQ was significantly improved in group 1 when using program 1 in unaided condition and to program 2. In the presence of background noise (SRTN) we can say that there was a trend towards a difference between both programs, as the statistical analysis revealed that the p-value was close to the acceptable limit. There were no significant differences between program 2 unaided and aided results.

SRTQ and SRTN were significantly improved in group 2 when using program 2 and unaided conditions and when using program 1 and aided conditions.

There was a significant difference between groups only in environments that favored communication; in this condition the most significant difficulty was found in group 2.

## DISCUSSION

The degree of high frequency hearing loss and the percentage index of speech recognition already suggested significant differences between groups 1 and 2 before we identified dead zones of the cochlea. We may say that a negative result for dead zones of the cochlea corresponded to the expected pattern in group 1, due to the audiometric sloping configuration but with no threshold differences over 50 dB obtained in successive octaves of tested frequencies, and the absence of thresholds over 90 dB NA at high frequencies (Table 3).^11^ We found a positive result for dead zones at high frequencies over 1500 Hz in group 2 in most cases (Table 4). Many patients reported hearing a different sound, similar to a hiss, when the pure tone frequency was associated with the dead zone.^12^

**Table 3:** Auditory thresholds (dB NA) and mean differences between masked thresholds and the noise level in group 1.

Frequencies (kHz)								
Subjects	0.5	0.75	1	1.5	2	3	4	
1	RE	32	28	34	36	36	56	60
	LE	32	32	30	34	38	44	58
2	RE	24	26	32	40	38	46	60
	LE	52	52	50	60	56	58	66
3	RE	18	22	20	28	60	58	54
	LE	20	20	20	38	56	62	52
4	RE	16	20	20	26	36	50	60
	LE	24	24	26	50	46	52	54
5	RE	18	26	30	46	48	54	60
	LE	26	26	32	44	48	50	58
6	RE	12	14	22	56	58	52	60
	LE	10	14	16	48	50	50	54
7	RE	12	8	10	36	50	54	54
	LE	18	24	36	36	54	54	60
8	RE	16	20	24	56	60	58	70
	LE	16	20	24	50	54	56	64
9	RE	24	34	50	50	50	54	70
	LE	28	32	42	42	42	48	60
10	RE	24	22	32	38	44	58	60
	LE	14	12	18	34	34	60	54
11	RE	32	38	38	50	50	58	66
	LE	12	24	30	56	50	62	80
12	RE	26	26	24	28	36	52	64
	LE	24	30	32	36	40	50	52
13	RE	6	10	18	38	52	54	56
	LE	10	46	50	50	52	56	56
14	RE	28	28	32	44	40	54	64
	LE	30	30	32	32	38	42	52
15	RE	18	14	8	42	54	54	60
	LE	38	28	52	54	52	64	64
Mean	RE	4.3	4.3	4.9	5.7	5.7	5.3	3.8
	LE	2.0	3.2	4.1	5.2	5.1	5.0	4.5

**Key:** RE - right ear; LE - left ear

**Table 4:** Auditory thresholds (dB NA) and mean differences between masked thresholds and the noise level in group 2.

Frequencies (kHz)								
Subjects	0.5	0.75	1	1.5	2	3	4	
1	RE	6	54	64	64*	66*	AR	AR
	LE	8	52	64	66*	86*	AR	AR
2	RE	6	28	52	64*	66*	64*	66*
	LE	8	14	46	70*	68*	64*	64*
3	RE	22	18	82*	82*	90*	AR	AR
	LE	20	26	74*	82*	80*	84*	82*
4	RE	42	52	60	86*	82*	80*	86*
	LE	34	52	54	80*	82*	76*	82*
5	RE	44	72	78*	78*	78*	94*	AR
	LE	38	48	74*	AR	AR	96*	AR
6	RE	88	86	98*	94*	94*	90*	80*
	LE	32	42	72*	92*	92*	AR	AR
7	RE	42	50	62	82	92*	AR	AR
	LE	60	62	68	80*	86*	82*	AR
8	RE	4	8	14	36	62*	58*	58*
	LE	4	12	16	58*	60*	58*	56*
9	RE	46	56	74	88*	AR	AR	AR
	LE	60	68	88*	AR	AR	AR	AR
10	RE	50	68	86*	AR	AR	AR	AR
	LE	46	62	68	74	80*	80	90*
11	RE	26	20	18	44	58*	98*	90*
	LE	10	8	10	32	56	82*	AR
12	RE	46	64	70*	70*	84*	74*	92*
	LE	46	70	74*	84*	82*	AR	AR
13	RE	42	66	70	80*	AR	AR	AR
	LE	50	62	72*	96*	AR	AR	AR
14	RE	18	28	30	40*	52*	64*	68*
	LE	20	18	28	30*	38*	62*	64*
15	RE	54	62	68	78*	84*	96*	94*
	LE	48	56	66	72*	86*	86*	84*
Mean	RE	3.9	2.6	6.4	13.2	15.1	18.5	19.5
	LE	3.9	4.6	8.5	13.2	14.8	17.2	19.5

**Key:** RE - right ear; LE - left ear; AR - absence of response at the maximum pure tone intensity (102 dB NA).

* presence of dead zone of the cochlea at the frequency tested.

**Figure 1:**
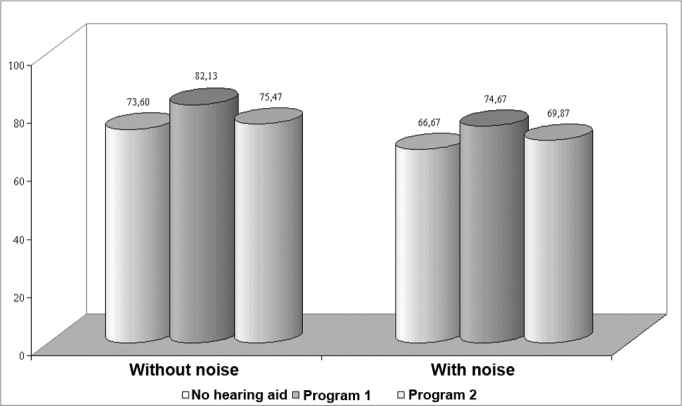
Chart showing the percentage index of speech recognition in quiet and with background noise in group 1.

The percentage index of speech recognition in group 1 showed a significant improvement when using hearing aids with program 1 (sound amplification in a wide frequency range from 100 Hz to 8000 Hz) compared to program 2 (restricted amplification from 100 Hz to 2560 Hz), both in quiet and in the presence of background noise (Figure 1). Thus, if there are no dead zones of the cochlea, high frequency information effectively contributes to speech intelligibility.^5^, ^7^, ^13^, ^14^

**Figure 2:**
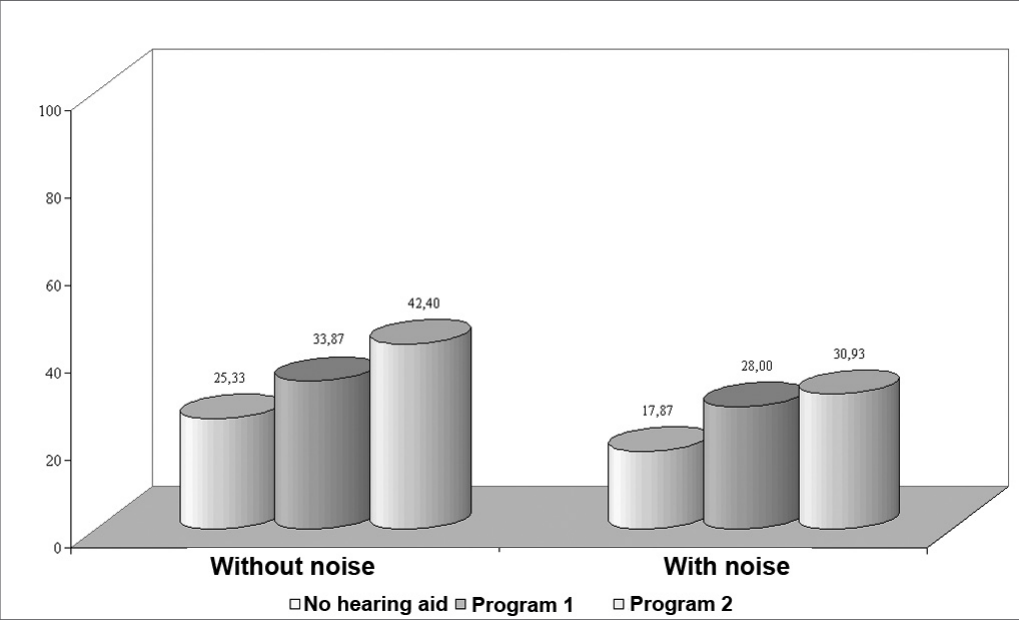
Chart showing the percentage index of speech recognition in quiet and with background noise in group 2.

The percentage index of speech recognition in group 2 was significantly improved by using hearing aids with programs 1 and 2 compared to the unaided condition, both in quiet and in the presence of background noise (Figure 2), although the highest benefit was seen with program 2. Sound amplification in a restricted frequency range - with a lower gain at frequencies in which hearing loss is most severe - favored information use where audibility is useful.^2^, ^5^, ^14^, ^15^

We may assume that patients with dead zones of the cochlea at high frequencies are used to a perception of filtered speech, as their hearing would operate as a low-pass filter. This could explain the benefit difference between each program for groups 1 and 2. Subjects with no dead zones, that effectively use high frequency information, are more affected by removal of these high frequencies.^14^

Comparing both programs, we observed that group 2 subjects reported increased clarity of sound and absence of hissing with program 2. We believe that the presence of dead zones at high frequencies reduce sound distortion by not amplifying those frequencies. Vibration generated in a dead zone is detected by another region; little useful information, therefore, is transmitted from the affected site. Furthermore, when the typical frequency of a region is different from that of the stimulus, detection of intense sound corresponding to these regions is altered.^6^

**Figure 3:**
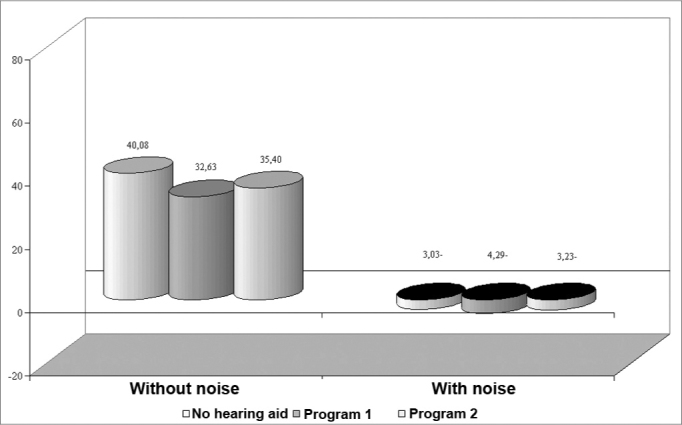
Chart showing the sentence recognition threshold in quiet (SRTQ) and with background noise (SRTN) in group 1.

Group 1 subjects performed better in the sentence recognition threshold test in quiet and in the presence of background noise when using program 1 compared to program 2 (Figure 3). There was no significant performance difference in program 2 with or without hearing aids in the presence of background noise; in this condition, program 2 offered practically no benefit. Once again, these results may be associated with the use of high frequency information to attain speech intelligibility.

**Figure 4:**
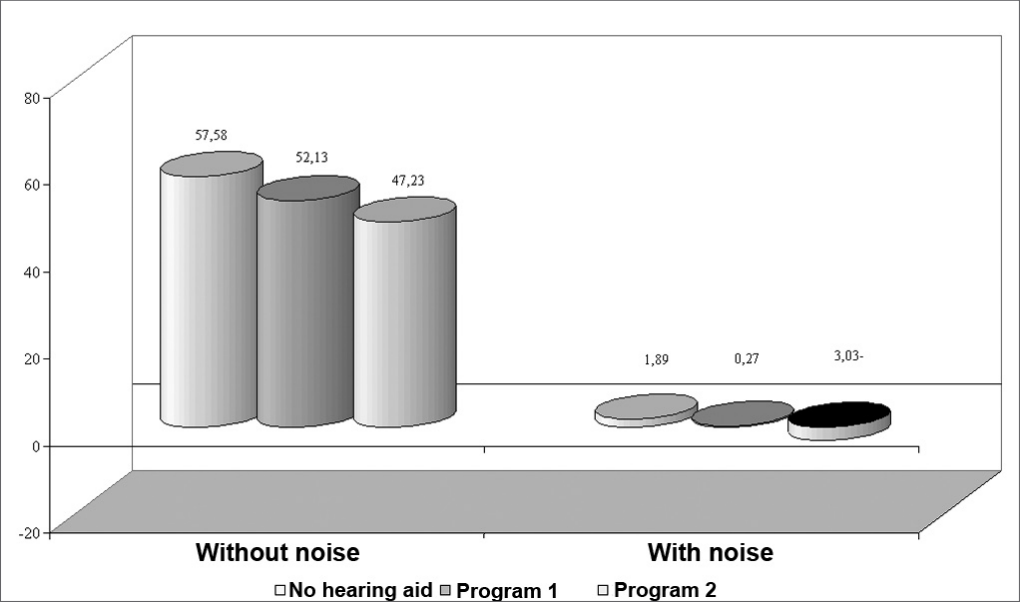
Chart showing the sentence recognition threshold in quiet (SRTQ) and with background noise (SRTN) in group 2.

Sentence recognition thresholds in quiet and in the presence of background noise (Figure 4) for group 2 benefited from both programs, where benefits from program 2 were more significant than those from program 1. The etiology and the time during which adequate auditory stimuli at high frequencies were absent should be taken into account when using high frequency amplification for conditions of marked hearing loss at these frequencies.^5^ Ten subjects with dead zones (66.7% of group 2) had a history of hearing loss for ten years or more, a relatively long period of absent auditory stimuli, which may have contributed to improved results with amplification of a reduced frequency range.

**Figure 5:**
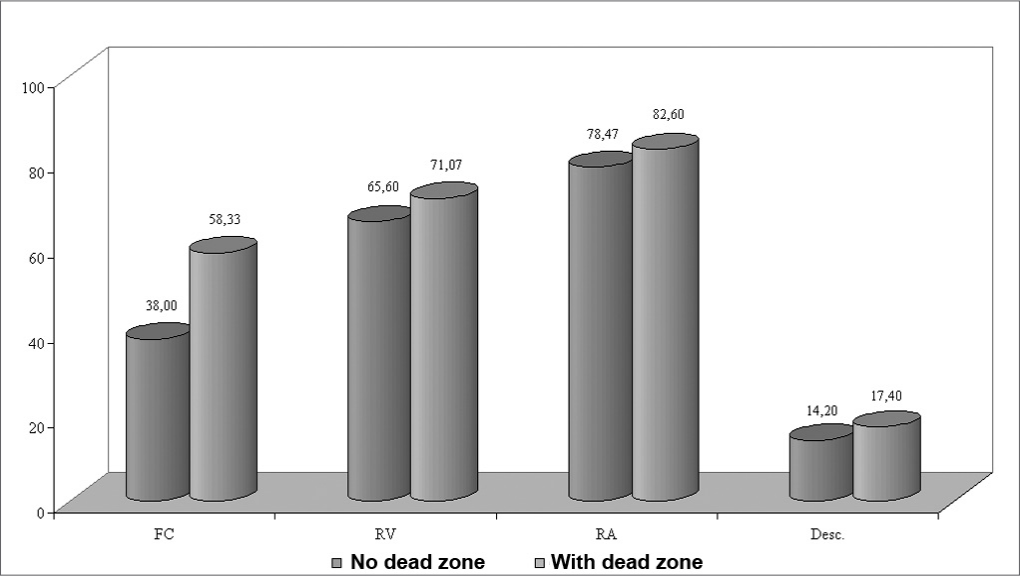
Chart showing the communication difficulty (%) in various sound environments and aversiveness to sounds according to the APHAB questionnaire in groups 1 and 2.

The APHAB questionnaire (Figure 5), used to compare both groups, revealed that there was a significant difference between groups only in the ease of communication environment, where group 2 had more communication difficulties. This result confirms the less favorable performance of group 2 in speech tests. This performance is related to the severity of hearing loss and the presence of dead zones of the cochlea.

Over half of group 2 subjects were going through hearing aid selection procedures just by taking part of this study, as previous adaptation attempts had been unsuccessful. In another paper the superior percentage index of speech recognition in patients with no dead zones of the cochlea was related to a higher acceptance rate of hearing aids (94.1%). In the presence of dead zones the acceptance rate was 21.4%. During the tests patients in group 2 reported improved sound quality with program 2, and that they wished to attempt adaptation once again.

Observing the region in which auditory thresholds were preserved most in group 2, we could question whether amplification of sounds corresponding to this area could truly benefit these patients. We know that there is also loss of low frequency phase/synchronization components together with loss of high frequency information.^3^, ^17^ Thus, gain at these frequencies is very important. We believe that the strategy of limiting sound amplification in affected areas where amplification would offer little benefit is the most adequate choice for the auditory rehabilitation of patients with dead zones of the cochlea.

## CONCLUSION

Based on a critical analysis of our results, we reached the following conclusions:

1. In the absence of dead zones of the cochlea, improved speech recognition performance is reached with amplification over a wide frequency range.
2. In the presence of dead zones of the cochlea at high frequencies, sound amplification of a restricted frequency range, avoiding gain at high frequencies, leads to the best speech recognition performance.
